# Future anxiety and coping methods of nursing students during COVID-19 pandemic: A cross-sectional study

**DOI:** 10.1097/MD.0000000000028989

**Published:** 2022-03-04

**Authors:** Senay Cetinkaya, Tugba Todil, Mustafa Kara

**Affiliations:** a Department of Nursing, Çukurova University, Adana, Turkey; b Health College, Batman University, Batman, Turkey; c Afşin Health College, Kahramanmaraş Sütçü İmam University, Kahramanmaraş, Turkey.

**Keywords:** coping methods, COVID-19, nursing students, state–trait anxiety inventory, stress, stress coping scale

## Abstract

The aim of this research is to examine the methods of nursing students to deal with future anxiety and stress.

It is a cross-sectional survey conducted in Turkey with 291 students in Çukurova University of Faculty of Health Sciences, Kahramanmaraş Sütçü İmam University Health College and Batman University Health College Nursing Department in June 2020. Personal Data Form, Stress Coping Scale, State and Trait Anxiety Scale were sent online to students’ smartphones and/or e-mails as data collection tools and it was collected this way.

The mean age of the participants was 21.09 ± 2.02 (years). 78% of respondents were women. 48.4% of the participants were students of Çukurova University. It was determined that 201 (69.1%) of the participants isolated themselves during the pandemic. It was found that 171 students (58.8%) spent 23 to 24 hours at home, whereas 284 students (97.6%) spent time with their parents/siblings. 47.4% of respondents stated that they had spent the pandemic watching a series/film. 47.1% of respondents had good family relationships. 50.2% of respondents had good relationships with college friends. 74.9% of respondents said they were happy. Women's trait anxiety scale scores were higher than men's (*P* < .05). Men have higher problem-Oriented coping scores than women (*P* < .05). Significant differences were found in the Status Anxiety Scale scores and trait anxiety scale scores according to self-isolation status (*P* < .05). A significant difference was found in terms of state anxiety scale and trait anxiety scale according to happiness status (*P* < .05). The problem-based coping scores of those who were happy with the Stress Coping Scale were higher than those who were not happy (*P* < .05). The state anxiety scale of the students was 42.54, and the trait anxiety scale was 45.16.

Nursing students’ status and sustained Anxiety Scale scores were moderate. It is important for individuals to have good family and friends and to be happy in the process of the Corona Virus Disease pandemic.

## 1 Introduction

Corona Virus Disease (COVID-19) appeared in December 2019 in Wuhan, the capital of Hubei region, which is one of China's largest industry and trade centers.^[1–4]^ As a result of a disease that has not yet been identified, developed without a cause and does not respond to known treatments and vaccines,^[3]^ it is understood that the disease called SARS-CoV-2 is caused by a new corona virus, and the disease has soon turned into an epidemic.^[2,3,5]^ The disease started in China, spread to the rest of Asia, Europe, and soon spread to the continent of Australia, the America and all over the world. On March 11, 2020, the outbreak was declared a “pandemic” by the World Health Organization (WHO).^[3,5]^ The virus can be transmitted very quickly from person to person with its high contamination feature. At the end of 10 cycles formed by the transmission of all 3 people to the other 3 people, the disease factor is transmitted to 59 thousand people. The transmission rate of the virus grew in January, and virus cases began to be reported in all countries on a global scale.^[3,5]^ Approximately 1200 participants from nearly 100 countries convened to review the science, to establish what we know now that we did not know at the start of the pandemic, and to set priorities for the COVID-19 research agenda. Situation in numbers (by WHO Region) Total (new cases in last 24 hours) globally 16 114 449 cases (254 274) 646 641 deaths (5490).^[6]^

The epidemic has brought about a number of concerns in humans. People in the risk group in particular experienced a peak sense of anxiety when they saw the number of cases and deaths taking place in the country and all over the world, considering the possibility of becoming sick and/or dying. Death anxiety has also had significant consequences related to social life.^[3]^

The first corona virus case in our country was seen on March 11, 2020.^[7]^ On March 10, the International Federation of Red Cross and Red Crescent Societies, United Nations Children's Fund, and WHO issued a new guidance to help protect children and schools from transmission of the COVID-19 virus.^[8]^ Following March 13, 2020, a 3-week break was given to education at the Universities of the Higher Education Institution. This period was later extended and switched to distance education system. University students are in one of the most important periods of their lives. During the university education, many factors such as the city where the education takes place, socioeconomic level, relations in the university environment, housing problem affect students’ anxiety.^[7]^ Anxiety is expressed as an adaptive mechanism to cope with danger, a basic human sense, and a multifaceted state of emotion.^[3]^ Increased anxiety leads to a person not knowing what to do about their future and not being able to make decisions. It causes people to have unwarranted fears, such as thinking something bad is going to happen to them.^[9]^

Based on the anxiety being acute and chronic, Spielberger (1966) proposed and scaled up the concepts of state and trait anxiety.^[10]^ Accordingly, Spielberger defined the state anxiety as an emotional reaction caused by individuals interpreting special situations as threats. On the other hand, he defined persistent anxiety as his reaction to being anxious and stressed about the situations in which individuals are present.^[3]^

Because human beings are a social entity, the impact of the social environment in which they live is important on the individual. Family is the first among them, because it is seen as a variable with higher power than other circles in the student's development of a healthy relationship. The demands of the family, expectations, life, attitude, and the number of siblings are considered as some of the important factors in the formation of anxiety in the student. Most of the anxiety in the students consists of the anxiety of failing to meet the high expectations of the parents in their school success.^[11,12]^

Academic stress is the unpleasant psychological situations that arise due to the educational expectations of parents, teachers, peers and family members, and the pressure of parents for academic success; the current education and examination system, assignment load and other factors clearly present an unpleasant situation for students.^[13]^ Nursing education is a planned education system consisting of complementary theoretical and practical departments. Clinical applications form an important part of Nursing Education. Clinical training enables students to integrate theoretical knowledge with practice and learn by doing and living in the real environment.^[12]^ During the process of the COVID-19 pandemic, nursing students remained out of clinical practice. Theoretical applications are continued with distance education system.

Many of the negative situations that individuals experience in their lives cause them to worry about their lives and to look at life more negatively.^[9]^ The uncertainty of the future is also a cause for concern. The lack of clear information about the positive or negative future in the person's mind prepares the ground for the development of future anxiety. Future anxiety is a condition that leads to pressure, stress, and despair on the young population.

Selye, one of the leading scientists interested in the subject of stress, described stress as “a general response of the individual to various environmental stressors”.^[14]^ Coping with stress is defined as the cognitive and behavioral efforts that individuals produce themselves when faced with stressful situations and develop to overcome the demands that come from their environment. The efforts of Folkman and Lazarus to cope in the stress-coping model were grouped under 2 groups, problem-oriented and emotion-oriented. Problem-oriented coping involves activities aimed at eliminating the threatening event or reducing its impact. Emotion-based coping, another approach to dealing with stress, includes activities such as neglecting the reality of the situation, avoiding the problem, and sharing negative emotions in order to reduce the effect of this stimulant, rather than struggling with the stress-inducing stimulant.^[15]^ The main purpose of dealing with stress is to eliminate the factors that negatively affect a person's life and productivity by reducing the amount of stress.^[16]^

Corona Virus Disease (COVID)-19 epidemic has become the main stress factor affecting the whole world in a short time due to its rapid spread due to the nature of the virus and its severe clinical course. Factors such as negative and distressing news about media and other communication tools related to the pandemic process, and the agenda being busy with the pandemic increase personal and mass stress and cause panic. After the first incident of corona virus in our country, schools were first vacated and then distance education was started. In this process, restrictions such as curfew restrictions and intercity travel restrictions came into our lives. These uncertainties were thought to affect students who continue their education as well as everyone else. In this context, the aim of the research is to examine the methods of coping with future anxiety and stress experienced by nursing students during this process.

## 2 Method

The research is a cross-sectional type survey conducted between June 1 and 30, 2020. The population of the study is students of Çukurova University Faculty of Health Sciences, Kahramanmaraş Sütçü İmam University AfşinHealth College and Batman Üniversity of Health College Nursing Department. The study took place with 291 students who voluntarily agreed to participate. When the research was carried out, students continued their education and training in their hometown with distance education.

### 2.1 Ethical approval

The research was carried out after obtaining approval from the Ethics Committee of Çukurova University and its official permission for application in related institutions. The research was in online format. Information about the study was given at the beginning of the survey link, which was sent to the students’ mobile phones and/or computers via e-mail. It was stated that the students were free to participate in the study or not. The questionnaire was applied as a result of their informed consent.

## 3 Data collection tools and equipment

Personal Information Form, Stress Coping Scale, State and Trait Anxiety Scales were used as data collection tools. These forms were converted to online format and students were asked to participate online by sending a survey link to their smartphones and/or e-mails.

### 3.1 Personal information form

The personal information form created by the researchers consists of 18 questions about socio-demographic characteristics and corona virus.

### 3.2 Stress coping scale

Scale items are arranged in simple sentences. Positive and negative statements are included on the scale in order to bring down the pattern response tendency. A. Sibel Türküm developed the scale in 2002. The scale was prepared in the form of a 5-point Likert-type scale and consists of 23 items. The score ranges of the options are as follows: “absolutely appropriate” = 5, “quite appropriate” = 4, “Neutral” = 3, “slightly inappropriate” = 2, “absolutely inappropriate” = 1. The participant was asked to mark only one of the options stating how well the situation expressed itself. Based on the data obtained from 110 university students, the correlation coefficient for the whole scale is (r = .85). Correlation coefficients for subscales were found as (r = .68) for seeking social support, (r = .71) for addressing the problem, and (r = .67) for avoiding trouble.^[15]^

Sub Scales:

- Avoidance Subscale: 8 items (8-40 points)
  Item Numbers: 1, 3, 11, 14, 15, 19, 21, 22
- Problem-Oriented Coping Subscale: 8 items (8-40 points)
  Item Numbers: 2, 5,6, 7, 8, 9, 12, 16
- Social Support Subscale: 7 items (7-35 points)
  Item Numbers: 4, 10∗, 13, 17∗, 18, 20∗, 23

(∗ Marked items will be scored in reverse).

In order to avoid the social desirability that may arise when filling the tool, the title is given as “Lifestyle Scale” and the name of the scale is obscured.^[15]^

### 3.3 State and trait anxiety scale

State – Trait Anxiety Inventory was developed by Spielberger et al,^[10]^ (1970), It was translated into Turkish in 1985 by Necla Öner and LeCompte and they provided the validity and reliability of the study. In the Turkish version of the scale, the reliability coefficients determined by alpha correlations were determined as .83 to .92 for the state anxiety scale and .83 to .87 for the trait anxiety scale. In the study, the state anxiety scale Cronbach alpha coefficient was .61 and the trait anxiety scale Cronbach alpha coefficient was .68. The State Anxiety Scale (STAI-1) reveals how the person feels at certain times and under certain conditions. The Trait Anxiety Scale (STAI-2) determines how the individual feels, unlike the environment and conditions in which the individual is located. In the State and Trait Anxiety Scale, there are 40 suggestions that individuals can use to express their feelings. Depending on how the person feels and the severity of his/her feelings, he/she should choose one of the following options: “None”, “A little”, “Quite”, “Completely” The State Anxiety Scale is composed of accurate propositions (3, 4, 6, 7, 9, 12, 13, 14, 17, 18) and inverted propositions (1, 2, 5, 8, 10, 11, 15, 16, 19, 20). The Trait Anxiety Scale is composed of direct propositions (22, 23, 24, 25, 28, 29, 31, 32, 34, 35, 37, 38, 40) and inverted propositions (21, 26, 27, 30, 33, 36, 39). When the reverse propositions expressing positive feelings are scored during the evaluation, those with a weight value are converted to 4 and those with 4 weight value are converted to 1. When expressing negative emotions, responses of 4 values in direct expressions indicate high values of anxiety. In inverted propositions, the responses at 4 are low and the responses at 1 indicate high anxiety. The total score of the direct propositions expressing negative emotions and the reverse propositions expressing positive emotions are retransmitted and the total score varying between 20 and 80 is obtained separately from the 2 scales. Another way to calculate scores is to deduce the total weighted score of inverse propositions from the weighted score collected for direct statements. A predetermined and unchanging value is added to the number found. The unchanged value in the state anxiety scale is 50, and the unchanged value in the trait anxiety scale is 35. The most recently found value reveals the individual's anxiety score. A high score indicates a high anxiety level, a low score indicates a low anxiety level. It is stated that if the total anxiety is over 60, it needs professional help. In addition, the scale's score ranges are used. The score ranges are as follows:

0-40 points: No anxiety,
41-60 points: Mild anxiety,
61 > points: Severe anxiety.^[17]^

## 4 Evaluation of data

Statistical analysis was performed using a package program called SPSS (demo package program). Frequency tables and descriptive statistics were used to interpret the findings. Test results were evaluated at .05 significance level (*P* < .05).

Parametric methods were used for measurement values suitable for normal distribution. According to parametric methods, “Independent Sample-*t*” test (T-table value) was used to compare the measurement values of 2 independent groups and “ANOVA” test (F-table value) method was used to compare the measurement values of 3 or more independent groups. The Tukey test was performed for binary comparisons of variables with significant differences for 3 or more groups, taking into account the homogenous variances.

Nonparametric methods were used for measurement values that were not suitable for Normal distribution. The “Mann–Whitney *U*” test (Z-table value) was used to compare the measurement values of 2 independent groups and the “Kruskal–Wallis H” test (χ^2^-table value) method was used to compare the measurement values of 3 or more independent groups according to nonparametric methods. Bonferroni correction was applied for binary comparisons of variables with significant difference for 3 or more groups.

Spearman correlation coefficient was used to examine the relationship between measurement values that do not have normal distribution.

An example for examining differences:

In variables that have significant differences for 3 or more groups, there are expressions such as “ [1-2,3]” in binary comparisons. [1-2,3] The meaning of this expression is that there is a significant difference between 1 and 2 and between 1 and 3.

## 5 Results

One hundred twenty-five students (43.0%) were determined to be in the 20 to 21 age group and the average age of all students was determined as 21.09 ± 2.02 (years). It was determined that 227 students (78.0%) were women, 114 of them (39.1%) had 3-4 siblings, 141 of them (48.4%) studied at Çukurova University and 201 of them (69.1%) isolated themselves during the pandemic. It was determined that 171 students (58.8%) spent 23 to 24 hours at home, 284 of them (97.6%) spent time with their parents/siblings and 158 of the students (54.3%) were born in the province (Table 1).

**Table 1:** Distribution of findings related to students.

Variable (n = 291)	n	%
Age groups $\left[\bar{\text{X}}\pm \text{S}.\text{S}.\to 21.09\pm 2.02(\text{year})\right]$		
19 and below	58	19.9
20-21	125	43.0
22 and above	108	37.1
Gender		
Boys/men	64	22.0
Girls/women	227	78.0
Number of siblings		
2 and below	64	22.0
3-4	114	39.1
5-6	68	23.4
7 and above	45	15.5
University		
Batman	59	20.3
Çukurova	141	48.4
Kahramanmaraş Sütçü İmam	91	31.3
Isolation		
Yes	201	69.1
No	16	5.5
Partially	74	25.4
Time spent at home (d/h)		
Less than 10 h	9	3.1
10-18	28	9.6
19-22	83	28.5
23-24	171	58.8
People living with him/her		
Mother, father, sister	284	97.6
Friend	5	1.7
Grandmother, grandfather	2	0.7
Birthplace		
Province	158	54.3
District	105	36.1
Village	28	9.6

It was determined that 242 students (83.2%) had an elementary family structure, 176 of the students’ (60.5%) income was equal to their expenses, 121 of them (41.6%) had a grade point average of 2.50 to 2.99 and 112 of the students (38.5%) were in the 3rd grade. It was determined that 38 students (47.4%) spent pandemic process by watching TV series/movies, 137 students (47.1%) had good family relationships, 146 of them (50.2%) had good relationships with their university friends and 218 of them (74.9%) were happy (Table 2).

**Table 2:** Distribution of students’ findings.

Variable (n = 291)	n	%
Family type		
Elementary	242	83.2
Extended	36	12.3
Broken	13	4.5
Income level of the family		
Income is less than expenses	78	26.8
Income is more than expenses	37	12.7
Income and expenses are equivalent	176	60.5
GPA		
<2,00	6	2.0
2.00-2.49	48	16.5
2.50-2.99	121	41.6
≥3.00	116	39.9
Grade		
1	87	29.9
2	42	14.4
3	112	38.5
4	50	17.2
Spending time during a pandemic		
By watching TV series/movies	138	47.4
By reading books	44	15.2
Studying	106	36.4
Touring	3	1.0
Level of family relationships		
Very good	77	26.4
Good	137	47.1
Middle	73	25.1
Bad	4	1.4
Relationship level with university friends		
Very good	78	26.8
Good	146	50.2
Middle	65	22.3
Bad	2	0.7
State of happiness		
Yes	218	74.9
No	73	25.1

There is no statistically significant difference in terms of state anxiety scale, trait anxiety scale, stress coping scale, avoidance, problem-oriented coping and social support scores by age groups (*P* > .05) (Table 3).

**Table 3:** Comparison of situational/trait anxiety scale and stress coping scale scores based on the students’ findings.

		State anxiety		Trait anxiety		*Avoidance*		*Problem-oriented*		*Social support*	
Variable (N = 291)	n	$\bar{\text{X}}\pm \text{S}.\text{S}.$	Median [IQR]	$\bar{\text{X}}\pm \text{S}.\text{S}.$	Median [IQR]	$\bar{\text{X}}\pm \text{S}.\text{S}.$	Median [IQR]	$\bar{\text{X}}\pm \text{S}.\text{S}.$	Median [IQR]	$\bar{\text{X}}\pm \text{S}.\text{S}.$	Median [IQR]
**Age groups** 19 and below20-2122 and above	58125108	43,24 ± 10,3842,36 ± 10,3142,38 ± 11,33	42,0 [12,3]44,0 [17,0]42,0 [15,0]	46,37 ± 8,4545,34 ± 9,6444,29 ± 9,03	42,0 [15,0]45,0 [12,5]44,0 [11,8]	27,48 ± 5,5027,74 ± 5,5528,12 ± 5,26	28,0 [6,0]29,0 [6,5]28,0 [6,0]	30,53 ± 5,6131,58 ± 6,1431,61 ± 5,87	31,0 [4,8]32,0 [6,0]32,0 [7,0]	21,19 ± 3,2520,98 ± 3,0521,20 ± 2,74	21,0 [3,3]21,0 [4,0]21,0 [3,0]
**Statistical analysis** ^∗^ **Probability**		χ^2^ = 0,246 *P* = ,884		χ^2^ = 2,104 *P* = ,349		χ^2^ = 0,282 *P* = ,868		χ^2^ = 2,946 *P* = ,229		χ^2^ = 0,350 *P* = ,839	
**Gender** MaleFemale	64227	41,78 ± 12,5542,76 ± 10,11	41,5 [17,0]42,0 [15,0]	41,29 ± 8,2446,25 ± 9,17	40,0 [11,0]46,0 [12,0]	28,33 ± 5,6627,66 ± 5,36	28,0 [8,0]28,0 [6,0]	33,30 ± 5,2130,84 ± 6,02	34,0 [8,0]31,0 [6,0]	20,89 ± 2,9721,16 ± 2,98	21,0 [2,0]21,0 [3,0]
**Statistical analysis** **Probability**		t = –0,645 *P* = ,519		Z = –3,876 ***P*** **=** **,000**		Z = –0,516 *P* = ,606		Z = –3,108 ***P*** **=** **,002**		Z = –0,672 *P* = ,502	
**Isolation** Yes [1]No [2]Partially [3]	2011674	41,14 ± 10,3549,56 ± 11,4944,84 ± 10,54	41,0 [15,0]50,0 [10,5]45,0 [13,5]	44,32 ± 9,4848,00 ± 8,6646,84 ± 8,21	44,0 [12,5]48,5 [11,0]46,5 [9,0]	27,66 ± 5,3427,81 ± 6,8328,30 ± 5,34	28,0 [6,0]29,5 [8,3]29,0 [5,3]	31,79 ± 5,7729,75 ± 6,8630,64 ± 6,11	32,0 [6,0]32,0 [6,3]31,0 [5,0]	21,00 ± 3,0621,50 ± 1,5521,30 ± 2,96	21,0 [4,0]21,0 [1,0]21,0 [2,3]
**Statistical analysis** **Probability** **Difference**		F = 7,192 ***P*** **=** **,001** **[1-2,3]**		χ^2^ = 6,244 ***P*** **=** **,044** **[1-2]**		χ^2^ = 1,838 *P* = 0,399		χ^2^ = 3,215 *P* = 0,200		χ^2^ = 0,128 *P* = 0,938	
**Income level of the family** Income is less than expensesIncome is more than expensesIncome and expenses are equivalent	7837176	44,67 ± 10,5042,16 ± 12,2741,68 ± 10,34	44,5 [10,5]41,0 [20,5]42,0 [15,8]	45,53 ± 8,2544,11 ± 9,6245,22 ± 9,52	45,0 [11,0]46,0 [13,0]45,0 [12,0]	27,77 ± 5,9527,92 ± 4,5627,84 ± 5,37	28,0 [7,0]29,0 [4,5]28,5 [6,0]	31,32 ± 5,9731,22 ± 5,6131,44 ± 6,01	32,0 [5,0]31,0 [6,5]32,0 [6,8]	21,35 ± 3,1121,45 ± 2,7120,92 ± 2,96	21,0 [3,0]21,0 [3,0]21,0 [4,0]
**Statistical analysis** **Probability**		F = 2,154 *P* = ,118		χ^2^ = 0,362 *P* = ,835		χ^2^ = 0,290 *P* = ,865		χ^2^ = 0,205 *P* = ,903		χ^2^ = 1,563 *P* = ,458	
**Pandemic process** By watching TV series/moviesBy reading booksStudying	13844106	43,09 ± 11,6441,20 ± 8,7442,33 ± 9,95	42,0 [17,0]43,0 [8,0]42,0 [13,5]	45,72 ± 9,8544,50 ± 7,1444,75 ± 9,15	46,0 [11,3]44,0 [10,8]44,0 [12,0]	27,40 ± 5,9327,98 ± 5,8628,29 ± 4,54	27,5 [7,0]28,5 [6,8]29,0 [5,0]	30,86 ± 6,6231,89 ± 5,2431,75 ± 5,24	31,0 [7,3]32,0 [6,0]32,0 [5,0]	21,11 ± 2,9520,77 ± 3,0621,16 ± 2,98	21,0 [3,0]21,0 [3,8]21,0 [3,0]
**Statistical analysis** **Probability**		F = 0,549 *P* = ,578		χ^2^ = 1,345 *P* = ,510		χ^2^ = 1,785 *P* = ,410		χ^2^ = 0,639 *P* = ,726		χ^2^ = 0,579 *P* = 0,749	
**State of happiness** YesNo	21873	41,15 ± 10,2746,70 ± 10,89	41,5 [15,0]46,0 [13,0]	43,42 ± 8,2750,37 ± 9,86	44,0 [11,0]49,0 [13,5]	28,13 ± 5,0226,95 ± 6,44	28,0 [6,3]27,0 [6,5]	31,91 ± 5,3329,79 ± 7,25	32,0 [5,3]30,0 [8,5]	21,33 ± 2,8820,42 ± 3,16	21,0 [3,0]21,0 [2,5]
**Statistical analysis** **Probability**		Z = –3,801 ***P*** **=** **,000**		t = –5,913 ***P*** **=** **,000**		Z = –1,484 *P* = ,138		Z = –2,156 ***P*** **=** **,031**		Z = –1,814 *P* = ,070	

∗ “Independent Sample-*t*” test (T-table value) was used to compare the measurement values of 2 independent groups in the Normal distribution data; “ANOVA” test (F-table value) statistics were used to compare 3 or more independent groups. The “Mann–Whitney *U*” test (Z-table value) was used to compare the measurement values of 2 independent groups in data that did not have Normal distribution; “Kruskall-Wallis H” test (χ^2^-table value) statistics were used to compare 3 or more independent groups.

There are no statistically significant differences in terms of state anxiety Scale, stress coping scale, avoidance and social support scores by gender (*P* > .05) (Table 3).

Statistically significant difference was determined in terms of trait anxiety scale scores by sexes (Z = –3,876; *P* = ,000). Women's trait anxiety scale scores were statistically significantly higher than men (Table 3).

A statistically significant difference was found in terms of coping with stress scale and problem-oriented coping scores by gender (Z = –3,108; *P* = ,002). Men's problem-oriented coping scores are statistically significantly higher than women's (Table 3).

Statistically significant difference in state anxiety scale scores was determined based on self-isolation (F = 7,192; *P* = ,001) (Table 3). In order to determine which group the significant difference was due, Tukey binary comparisons were made taking into account homogeneity of the variances and statistically significant differences were determined between self-isolating and non-isolating and partially isolating. The situationally anxiety scale scores of those who are not isolating themselves and those who are partially isolating are statistically significantly higher than those who are self-isolating.

Statistically significant difference in trait anxiety scale scores was determined based on self-isolation (χ^2^ = 6,244; *P* = ,044) (Table 3). As a result of Bonferroni-corrected binary comparisons to determine which group the significant difference was due to, statistically significant differences were determined between self-isolating and non-isolating groups. Trait anxiety scale scores of those who do not self-isolate are statistically significantly higher than those who self-isolate.

There are no statistically significant differences in the scale of coping with stress in terms of avoidance, problem-oriented coping and social support scores, according to the state of self-isolation (*P* > ,05).

There are no statistically significant differences in the state anxiety scale, trait anxiety scale, stress coping scale, avoidance, problem-oriented coping, and social support scores according to the income level classes of the family (*P* > .05) (Table 3).

There are no statistically significant differences in state anxiety scale, trait anxiety scale, stress coping scale, avoidance, problem-oriented coping, and social support scores according to the state of the pandemic process (*P* > ,05) (Table 3).

Statistically significant difference was determined in terms of state anxiety Scale scores compared to happiness status (Z = –3,801; *P* = ,000) (Table 3). The state anxiety Scale scores of those who are happy are statistically significantly lower than those who are not happy.

Statistically significant difference was determined in terms of trait anxiety scale scores relative to happiness status (t = –5,913; *P* = ,000) (Table 3). The trait anxiety Scale scores of those who are happy are statistically significantly lower than those who are not happy.

Statistically significant difference was determined in terms of stress coping scale, and problem-oriented coping, according to happiness status (t = –5,913; *P* = ,000) (Table 3). The stress-coping scale and problem-Oriented coping scores of happy individuals are statistically significantly higher than those who are not happy.

There are no statistically significant differences in stress coping scale avoidance and social support scores relative to happiness status (*P* > .05) (Table 3).

There is no statistically significant correlation between State anxiety Scale scores and stress coping scale, avoidance and social support scores (*P* > .05) (Table 4).

**Table 4:** Examining the relationships between situational/trait anxiety scale and stress coping scale scores.

		Anxiety scale	
Correlation^∗^ (n = 291) Stress coping scale		Situational	Continuous
*Avoidance*	rp	0,0040,948	0,0140,808
*Problem-oriented coping*	rp	−**0,253** **0,000**	−**0,334** **0,000**
*Social support*	rp	−0,0190,750	−0,0550,349

∗ The “Spearman” correlation coefficient was used in the study of the relations of 2 quantitative variables that do not have a normal distribution.

Negative, weak and statistically significant correlation was found between Status Anxiety Scale scores and stress coping scale problem-oriented coping scores (r = –0,253; *P* = ,000) (Table 4). As the Status Anxiety Scale scores increase, the stress coping scale problem-oriented coping scores decrease. Likewise, as the State anxiety Scale scores decrease, the stress coping scale problem-oriented coping scores increase.

There is no statistically significant association between sustained Anxiety Scale scores and stress coping scale, avoidance and social support scores (*P* > .05) (Table 4).

Negative, weak and statistically significant correlation was found between trait anxiety scale scores and stress coping scale and problem-Oriented coping scores (r = –0,334; *P* = ,000) (Table 4). As trait anxiety scale scores increase, the stress coping scale, problem-oriented coping scores will decrease. Likewise, as trait anxiety Scale scores decrease, the stress coping scale, problem-driven coping scores will increase. The distribution of the findings on the scales was given in Table 5.

**Table 5:** Distribution of findings related to the scales.

	Findings			
Scales	Average	Standard deviation	Median	Min-Max
*State anxiety scale*	42,54	10,68	42,0	20,0-77,0
*Trait anxiety scale*	45,16	9,19	45,0	23,0-77,0
*Avoidance*	27,83	5,42	28,0	8,0-40,0
*Problem-oriented coping*	31,38	5,93	32,0	8,0-40,0
*Social support*	21,10	2,97	21,0	10,0-29,0

## 6 Discussion

It was determined that 284 (97.6%) of nursing students spent time with their parents/siblings (Table 1). One of the most important tools in coping with stress is known to be the person's family life. Family is seen as one of the most important ways to deal with stress, as it provides social support to the person.^[3]^ While understanding and promoting spiritual well-being, it is necessary to determine what affects people's well-being.

Another important feature of psychological resilience is that it can be learned. It is known that the state of well-being is related to age, period, environment, mental and spiritual posture. The most up-to-date definitions of well-being include multi-layered States of emotion.

74.9% of the students stated that they were happy (Table 2). The trait anxiety scale scores of those who are happy are statistically significantly lower than those who are not happy (Table 3).

Stress is basically a condition that can be experienced by all people regardless of age, gender, race, and other aspects of life. Stress is considered to be inseparable from human life and can be said to be a part of life.^[13]^

Research shows that stress causes the onset and increase of many diseases. It affects a wide range of human health, from internal distress to disruption of the body's immune system. Stress can upset the entire balance. It is your brain that provides the chemical balance in the body, and your brain drives your thoughts. Everything arises from thought: Pessimistic thought also negatively affects chemical balances in the body. Therefore, the power of positive thinking should not be ignored.^[18]^

By opening up to the society with the friendship relationships that young people will establish, help and emotional exchange by entering into friendship bonds. Being accepted and liked by friends causes the young person to have self-confidence and respect, to consider himself valuable and thus to be more reliable and consistent in human relationships.^[9]^

One hundred forty-six (50.2%) of nursing students were determined to have good relationships with their college friends (Table 2). Friendships in school affect the individual's social attitudes, which include all attitudes and behaviors toward social life, and provide moral support individually.^[9]^

A significant difference was found in terms of scores of trait anxiety scale according to gender. Women's trait anxiety scale scores were significantly higher than men (Table 3). Sakaoglu et al,^[3]^ (2020) stated that gender was similar among the independent variables affecting the level of anxiety in their research. In research on the relationship between anxiety and gender, it was found that when psychiatric samples were taken, the level of anxiety in women was higher than in men.^[9]^

In the study, the constant anxiety level of women (46.25) was higher than that of men (41.29) (Table 3). Çakmak & Hevedanli (2005) found that the anxiety levels of female students (46.73) were higher than the anxiety levels of male students (43.43) in a study they conducted in 264 students attending Biology Department of Dicle University Ziya Gökalp Education and Science and Literature Faculties.^[9]^ In the studies of Sakaoğlu et al^[3]^ (2020), the average of trait anxiety score was 44.21 in women and 43.95 in men. In this respect, it is similar to our study. However, in the study of Sakaoğlu et al,^[3]^ this difference was not found to be statistically significant. In the study of Kula and Saraç (2016), similar to our study, it was concluded that gender is a factor that makes a difference on students’ anxiety scores, and it has been identified that the average of female students’ trait anxiety scores (44.44) is significantly higher than the average of trait anxiety scores of male students (41.37) (*P* < .05).^[11]^ This can be explained by the fact that women's general anxiety levels are higher than men's and women's focus on their emotions more than men.^[3]^ Anxiety also takes its origin from childhood years.^[9]^

Nursing students’ trait anxiety scale score was found 45.16 (Table 5). According to this result, we can say that university students experience moderate anxiety. In the study of Sakaoglu et al,^[3]^ (2020), mean trait anxiety score was found similar (44.16).^[3]^ Similarly, in Kula and Saraç (2016), students’ mean anxiety score was found to be at an average level of 43.41.^[11]^ Sakaoglu et al,^[3]^ (2020) stated that the anxiety scores of health workers were slightly higher during the pandemic period, given the previous anxiety scale studies on health workers. He interpreted this as being in line with the state of uncertainty caused by the pandemic.^[3]^

Health care and health workers, which are one of the most risky business lines in terms of Health and safety, are the ones where the current risk is raised to the highest level during the pandemic processes. These risks include physical, chemical, biological factors, and psychosocial structure arising from the working environment. In the most general sense, stress is assessed as an environmental factor and the combination of this environmental factor perceived by the individual.

As a matter of fact, Utami et al,^[13]^ found that the student self-efficacy status was low while the student stress was generally high in the April-May 2020 period during the epidemic of 2020 COVID-19. Self-efficacy is an extension of Social Cognitive Theory, which is also a learning process. Basically, this aspect is defined as a person's personal belief in their ability to effectively perform a particular task. Self-sufficiency can be used to predict the rise and/or fall of academic stress on students. Utami et al,^[13]^ (2020) stated that self-efficacy accounted for 22% of factors predicting academic stress, whereas 78% consisted of other variables not studied by this study.

Hasanah et al^[19]^ (2020) determined that they experienced mild anxiety in 79 students, mild stress in 23 students, and mild depression in 7 students in their quantitative research with a descriptive analytical approach with 190 students. It has shown that many of the psychological problems experienced by students in the online learning process are of concern. Student anxiety, stress, and depression are exacerbated by the presence of the COVID-19 epidemic through online learning methods.

The group of employees assessed by Occupational Safety and Health Administration in a veryhigh and high risk group for COVID-19 infection are health workers.^[3]^ In a cross-sectional study on general practitioners doctors working in Italy, the mental health of the Covid-19 pandemic was evaluated. In a study of 130 physicians, anxiety (13.43 ± 4.96 and 11.60 ± 5.53), no sleep (4.88 ± 3.53 and 4.84 ± 3.81), mental and quality of lifespan (4.78, *P* < .001) above was significant.^[20]^ Therefore, the trait anxiety score may have resulted in a moderate level in our study of nursing students. Due to the strong infectivity of COVID-19, the number of infected people rapidly increased in a short time. This caused significant collective fear and anxiety in society. Since there is no literature on this subject, we hope to contribute with our study.

Intense stress automatically leads to the formation of a number of physiological symptoms in the organism. Symptoms such as palpitations, shortness of breath, muscle tension, and novelties added to them in the later period, such as distraction, can reach a size that disrupts one's life, especially when it is very severe. Not knowing that these are of psychological origin may lead the person to seek various examinations and treatments. These symptoms can only be lost by reducing the level of anxiety.^[18]^

Persistent long-term anxiety can cause stress that interferes with daily activities. If not resolved it could lead to more serious psychological problems such as depression.^[19]^ In a study examining the attitudes toward suicide among university students in South Korea and the evidence on risk factors for suicide attempts; it is reported that depression, bipolar disorder, negative attitudes toward oneself, the complexity of life, anxiety and stress related to academic success, family problems and hopelessness are the main risk factors for suicide attempts.^[21]^

In the study conducted with students studying medicine; confirmed that chronic stress and anxiety have a negative effect on mental health and are associated with suicidal ideation in their students.^[22]^

The WHO has established that one million people die by suicide every year, with the impressive daily rate of a suicide every 40 second. The weightiest concern about suicidal behavior is how difficult it is for healthcare professionals to predict.^[23]^ Modern psychiatry needs a more careful assessment of suicide risk stratification and better interpretation of suicide risk with planning clinical and therapeutic interventions.^[24]^

It is important to continue to investigate the effects of a pandemic on students’ mental health so that its effects can be prevented or at least reduced. Regular screening of students’ mental health is recommended to identify students with psychological problems.

The roles of Academic Advisor lecturers, lecturers and the guidance and Counselling Service Unit in each higher education institution are able to produce programs that can help to increase self-sufficiency for students; because if the students are in a state of constant academic stress, this will definitely prevent the continuity of student studies.^[13]^

According to thinkers of Existential Philosophy, traumas are natural reasons for questioning life. Challenging life events lead people to struggles, resulting in some positive emotional, mental and behavioral changes. The question of how to develop psychological resilience is now once again important to business, education and policy makers, as the emotional and psychological problems of today's people have become an epidemic and become a serious public health problem.

Based on these results, it is predictable that the outbreak could psychologically affect many as it turns into a public health problem. Therefore, in order to combat the psychological aspects of the epidemic, psychosocial interventions of psychiatrists, nurses, psychologists and psychological counselors will contribute positively to the psychological resilience of individuals.

When we take a look at the strategies for developing psychological robustness laid out by the American Association of psychologists (APA, 2016), we can see once again that relatedness, the spirit of neighborhood and community, and being at peace with oneself are important. These strategies are listed as follows:

- Having good relationships with family members and the inner circle,
- Taking part in social groups,
- Accepting help when needed,
- Not to escape from problems that seem unbearable,
- Having realistic expectations in solving problems,
- Evaluating potential problems in advance,
- Considering unexpected possible situations as a natural part of life,
- Setting achievable goals,
- Not exaggerate the event outside the truth, even though it is painful,
- Increasing positive expectations about life,
- Paying attention to self-care,
- Doing enjoyable and relaxing activities.^[25]^

It is thought that psychological counseling and guidance services should be increased to help students have a healthy mental structure by reducing their anxiety levels. In addition, mothers and fathers are expected to be sensitive to their children and to monitor their biopsycho-social development, to find solutions to cognitive and affective problems with experts and teachers, which will positively affect the self-structure of the young people and decrease the level of anxiety.^[9]^

The activities and psychosocial support of multidisciplinary mental health professionals during the COVID-19 outbreak in Korea are examples of this situation. Hyun et al,^[26]^ (2020)'s review article describes the potential power of multidisciplinary teamwork during the COVID-19 disease outbreak crisis. Other professionals and organizations in Psychiatry, Psychology, Social Work and nursing have provided a variety of supportive activities, such as telephone counseling for the public. Psychosocial support during an outbreak is no less important than infection control.

Let's not forget that each individual feels differently and grows at different times. The important thing is not to feel alienated and alone in this process. The emotions, thoughts and knowledge we experience in our own world that make us feel good despite their difficulties are important. No matter the size or the smallness of these things, they are our reality and what is meaningful to us.

“Every epidemic deletes something old, starts a new life” approach is also valid for COVID-19. With this outbreak, it has been seen that no vital parameters will continue as before. If individuals do not underestimate the risk factors and create a lifestyle in accordance with scientific measures and universal general truths, the experience in this process can turn into positive gains.

## Author contributions

**Conceptualization:** Senay Cetinkaya, Tugba Todil.

**Data curation:** Tugba Todil, Mustafa Kara

**Formal analysis:** Mustafa Kara

**Funding acquisition:** Senay Cetinkaya, Tugba Todil, Mustafa Kara.

**Investigation:** Senay Cetinkaya, Tugba Todil.

**Methodology:** Senay Cetinkaya, Tugba Todil, Mustafa Kara.

**Resources:** Senay Cetinkaya, Tugba Todil, Mustafa Kara.

**Software:** Senay Cetinkaya, Tugba Todil.

**Supervision:** Senay Cetinkaya,

**Validation:** Senay Cetinkaya, Mustafa Kara.

**Visualization:** Senay Cetinkaya,

**Writing – original draft:** Senay Cetinkaya, Tugba Todil.

**Writing – review & editing:** Senay Cetinkaya.
